# A Comprehensive Review on the Chemical Constituents and Functional Uses of Walnut (*Juglans* spp.) Husk

**DOI:** 10.3390/ijms20163920

**Published:** 2019-08-12

**Authors:** Ali Jahanban-Esfahlan, Alireza Ostadrahimi, Mahnaz Tabibiazar, Ryszard Amarowicz

**Affiliations:** 1 Nutrition Research Center, Tabriz University of Medical Sciences, Tabriz 5166-15731, Iran; 2 Student Research Committee, Tabriz University of Medical Sciences, Tabriz 5166-15731, Iran; 3 Infectious and Tropical Diseases Research Center, Tabriz University of Medical Sciences, Tabriz 5166-15731, Iran; 4 Division of Food Sciences, Institute of Animal Reproduction and Food Research of the Polish Academy of Sciences, 10-468 Olsztyn, Poland

**Keywords:** by-products, functional applications, fruit, husk, walnut

## Abstract

The walnut (*Juglans* spp.) is an appreciated nut that belongs to the Juglandaceae family. The fruit includes four main parts: the kernel, the skin, the shell, and the green husk. It is widely cultivated due to its edible kernel. In walnut production centers, high amounts of the husk as an agro-forest waste product are produced and discarded away. Recently, it has been demonstrated that the walnut green husk could be valued as a source of different natural bioactive compounds with excellent antioxidant and antimicrobial properties. Regarding this respect, in this contribution, the current scientific knowledge on the antioxidant and antiradical activities, various identified and isolated individual chemical constituents, as well as the functional applications of the walnut husk with more emphasis on the Persian walnut (*Juglans regia* L.) are reviewed.

## 1. Introduction

In recent years, plant-based materials, and especially nuts, have received much consideration and interest [1,2,3]. Today, the utilization of such substances is an important issue because they contain valuable compounds that could be advantageous for obtaining various beneficial compounds [4,5,6]. In this regard, agricultural waste products have been widely investigated because they are available in large quantities and discarded away without any control, causing environmental pollution. Additionally, the requirement for the naturally derived chemicals in nutrition, food, cosmetics, pharmaceuticals, and industrial applications are increasing rapidly due to the fast growth of global demand and consumer awareness [7]. Walnuts are mainly cultivated in order to obtain the kernels, and other parts of fruit such as the shell and husk are produced as waste crops during the fruit harvesting and processing [8,9]. It has been well documented that in addition to the kernel [10,11,12,13,14,15,16,17,18,19,20,21], different parts of the tree and fruit, including the green young walnut fruit [22,23,24,25,26,27], husk [28,29,30,31,32,33], shell [8,34,35,36,37,38,39,40,41,42], skin [43,44], even bark [45,46,47,48,49,50,51], root [52], shoot [53,54,55,56], branch [57], and leaves [58,59,60,61,62,63,64,65,66,67,68,69,70,71,72,73,74,75,76,77] can be employed in different industries as low-cost materials [78]. Similar to other agricultural waste crops, the walnut husk has been comprehensively investigated to characterize its chemical constituents and define new applications for it. Hence, this review aims to discuss the recent scientific literature regarding the importance of the walnut, including the different parts of its fruit, with more emphasis on the husk, the antioxidant and antiradical activities of walnut husk extract, and the isolated and identified chemical constituents of walnut husk, as well as provide further details. Finally, the functional applications of the walnut husk in different fields such as industry, medicine, and food are highlighted, as well as other uses.

## 2. Walnuts

The Juglandaceae family comprises between seven and 10 genera and about 60 species distributed mainly in the Northern Hemisphere of both the Old and New World. *Juglans* is a plant genus of the Juglandaceae family, whose seeds are known as walnuts. This genus includes 21 species from Southeastern Europe to Japan [79]. In Juglandaceae genera, the secondary metabolites including tetralones, naphthoquinones, and diarylheptanoids are considered to be useful chemotaxonomic markers for the characterization of the Juglandaceae family species.

*Juglans mandshurica* Maxim. (*J. mandshurica* Maxim.) is a deciduous tree that is widely distributed in northeastern Asia. Besides its edible fruit, *J. mandshurica* Maxim’s green husk was also used in folk medicine because it shows antioxidant, antitumor, and antibacterial properties. *J. mandshurica* Maxim. has been reported to be rich in naphthoquinones, diarylheptanoids, and flavonoids, as well as their glycoside form [80,81].

Black walnut or *Juglans nigra* (*J. nigra*) is a deciduous tree that is known as the eastern black walnut. This tree is indigenous to eastern North America, where it is grown in places such as South Dakota in the United States [82]. The detachment of its fruit takes place around October, consisting of a husk, a hard shell, and a kernel. In general, the husk is discarded during processing, although it contains phenolic compounds with antioxidant and antimicrobial properties [83].

The Persian, English walnut—or *Juglans regia* L. (*J. regia* L.)—is a famous member of the genus that is broadly cultivated because of the commercial high added value of the seeds. *J. regia* L. is a relatively nutritious food that is rich in bioactive natural products. It is a crucial tree nut and is an integral part of Mediterranean nutrition. Besides its use in nutrition, *J. regia* L. is also used for medicinal purposes [84,85]. This widely spread deciduous tree grows natively and commercially in Europe, Asia, and the eastern and southern parts of United States for two primary purposes: quality timber and the nut containing the edible meat [86,87].

## 3. The Health Benefits of Walnuts

Nuts are described as a leading source of phenolic compounds with high antioxidant activity, particularly walnuts, pecans, pistachios, hazelnuts, almonds, and peanuts [28,88,89,90,91,92,93,94,95,96,97]. The nut of the Persian walnut is essential in human nutrition, because it contains the valuable kernel, which shows high nutritional capacity due to high levels of unsaturated fatty acids, digestible proteins, phenolic compounds, and dietary fiber [21,98,99]. According to the performed studies, among all the studied nuts and seeds, the Persian walnut shows the highest content of antioxidant compounds [43,100,101]. Similarly, in an investigation by Gunduc and El [102], they demonstrated that the kernel of *J. regia* L. contains the highest content of total phenolic compounds and antioxidant activity among 25 types of commonly consumed foods. It has been reported that the regular and adequate consumption of the walnut seed is associated with the reduction of the risk of some diseases such as cancer [20,103], cardiovascular symptoms [104,105,106,107,108,109,110,111,112,113,114,115,116], diabetes [117], and degenerative disorders [118,119]. Different parts of the walnut tree and its fruit, including the green husk as epicarp or mesocarp, hard shell of the nut (endocarp), dividing membranes of the kernel (pellicle), flower, root, trunk (bark and wood), branch, and leaf are comprehensively considered in order to use for different purposes. Green pericarps have been used in Chinese traditional medicine for their anti-cancer and antioxidant properties as well as for the treatment of pain, inflammation, and skin diseases [120]. The leaves of the different walnut species were traditionally used in several European countries to alleviate minor inflammatory skin disorders [121]. The walnut husk is an agro-industrial residue that is available after walnut farming and processing activities [122]. The walnut ripe fruit has been freshly consumed in confectionery applications, while the young form of the fruit is mainly served to produce liqueur [27]. This particular drink is made from green walnut fruit at an early stage of fruit development [26]. It has been evidenced that juglone is the active ingredient in walnut that can inhibit the key enzymes required for metabolism. Juglone is known as a crucial phenolic compound that is present in different parts of walnut [120]. It shows excellent biological activities, including antimicrobial effects, and is also reported to decrease the incidence of tumors in the small intestine of rats [123]. Recently, juglone has been receiving interest as a potent anti-cancer agent [124,125,126]. Pyrogallol is known as another phenolic compound that is described in Persian walnut [21]. As potent anti-cancer agents, the interaction properties of these natural chemicals with serum albumins have been comprehensively investigated [120,127].

## 4. Walnut Fruit

**Figure 1:**
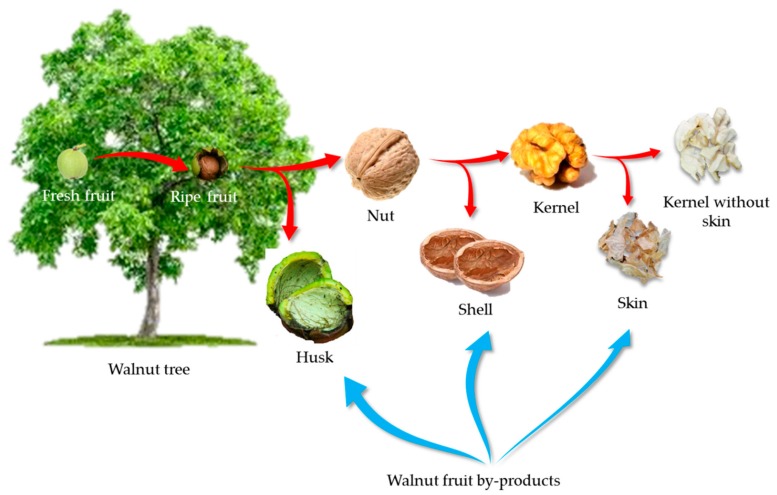
An unripe and young walnut fruit before its husk cracks and the ripe form of the fruit. Different parts of walnut fruit: kernel, skin, shell, and green husk. The shell and husk are the significant by-products of walnut fruit.

From the nutritional point of view, the walnut fruit is an essential part of the plant in the human diet, because it contains a valuable kernel. As illustrated in Figure 1, the walnut fruit is generally composed of four distinct parts. The green leathery outer layer is known as the husk or hull. When the fruit on the tree completely ripens, it cracks and is known as the nut after the manual separation of the husk. The shell is the name of the middle part of the fruit, which is a hard and inert layer with a light brown color surrounding the kernel. In order to release the kernel, the shell must be mechanically cracked. The kernel or meat is described as the edible part of the fruit, which is widely consumed by humans. Additionally, the kernel is covered by a thin light brown layer, which is known as the skin. In the case of other tree nuts such as almonds, this part of the nut contains high amounts of antioxidant compounds. Similar results have been reported for the walnut skin [16,28,43,44]. The antioxidant compounds in this part of the fruit are thought to act as protective agents against the deleterious effects of extreme UV radiation, bacterial, viral, and fungal contamination [8,90].

## 5. Walnut Fruit By-Products

The green husk and hard shell are the characteristic by-products of the fruit that are generated in walnut cultivation centers. Both of these agricultural waste products are closely linked and result from the production of the nut. The walnut husk and shell show different biomass characteristics in terms of content, use, accessibility, and distribution [8]. In the rural areas of some countries, the green husk and the hard shell are traditionally used as an energy source for heating purposes. Recently, different forms of utilization have been reported for the hard shell of the walnut, and thus, it has been subjected to further investigations [128]. Although the green husk of the walnut fruit is widely used in traditional medicine and current utilization in the industry has been reported, the obtained hard shell biomass from walnut fruit is more advantageous in terms of utilization when compared to the green husk [38,39,128]. For example, utilizing the walnut shell as filler in the preparation of polymer composites is one of the possible uses that has been recently described. The chemical composition of walnut shell fibers includes ash (3.4%), lignin (50.3%), hemicellulose (22.4%), and cellulose (23.9%). Due to the lower quantities of hygroscopic components and greater quantities of hydrophobic components in walnut shells compared to wood, polymer-based composite materials containing walnut shell fillers have significant commercial benefits in outdoor products requiring high environmental resistance, such as flooring or fencing [129,130,131,132,133].

## 6. Walnut Husk

The outer green thick layer of the walnut fruit is termed its husk, which is an abundant agricultural waste crop produced upon the harvesting of fruit and its processing. If this waste material is not adequately disposed of, it can cause environmental pollution [8]. However, it could be considered a valuable source of natural phenolic antioxidant or other beneficial compounds. Recently, the walnut husk has received increasing interest in modern pharmacology due to its excellent antioxidant activities, because it is a waste product that has been extensively used in traditional medicine for the treatment of skin diseases and the alleviation of the pain [134]. The main by-products derived from the walnut fruit are the walnut green husk and the hard shell. The walnut husk can be used as a natural dyeing source, such as juglone [29]. Juglone is a brown pigment with a chemical formula of 5-hydroxy-1,4-naphthoquinone, which occurs naturally in different parts of walnut trees such as the leaf, root, husk, and bark in the Juglandaceae family, especially in the Persian walnut [120]. Nowadays, the green husk as an agricultural by-product has a scarce utilization. Hence, the employment of husk as a source of phytochemicals or natural compounds with antioxidant and antimicrobial properties will enhance the value of the walnut cultivation, as well as define new usages for an agro-forest waste product, which is generated in high amounts [26,28,30,31].

## 7. Preparation of Walnut Husk Extract

The selection of a suitable solvent is a critical point during the extraction process and in the obtainment of extracts with high antioxidant activity that can be useful for the development and application of the green husk of the walnut. For example, the effects of different solvents (hexane, ethyl acetate, acetone, ethanol, methanol, and water) on the phytochemical content, including the total phenolic content (TPC), total flavonoid content (TFC), total condensed tannin content (TCTC), and the antioxidant activity of the green husk of the walnut were investigated. The authors showed that the extraction solvents that were tested significantly affected the phytochemical and antioxidant content of the green husk of the walnut. It has been found that the acetone, ethanol, and methanol extracts had the higher content of phytochemicals, and exhibited stronger antioxidant activities, followed by the ethyl acetate and water extracts, and the lowest for hexane extract [135]. Another study reported by Meshkini and Tahmasbi [136] determined the TPC, TFC, and TCTC of the Persian walnut husk extract, and evaluated its biological effects on the function of platelets. Their obtained results showed that the prepared acetone extract from walnut husk contained a high content of polyphenolic compounds with acceptable antioxidant activity.

The effect of various solvents—including water, ethanol, methanol, and 50% methanol and ethanol aqueous solutions—on the extraction yields and bioactive properties of extracts obtained from the walnut husk has also been reported [30]. The highest extraction yield was achieved with water, and the samples extracted with water/ethanol (1:1) demonstrated a high bioactive potential. The potential of the green husk of walnut was suggested as an economical source of antioxidants. Various extracts from the green fruits of the ‘Sibisel 44’ walnut cultivar were prepared and investigated for their TPC and TFC, different phenolic compounds content, antioxidant activity, and color components [23]. Extracts were prepared from the green husk of the walnut using three variants for evaluating the effects of solvent concentration and the addition of sugar on the properties of extracts. They detected 17 individual phenolics and quantified them with high-performance liquid chromatography (HPLC) in walnut green fruit extracts. They observed that 70% of ethanol was more efficient for the total phenolics and flavonoids extraction from green walnut fruits. They also found the highest concentration of phenolics in extracts prepared by 70% ethanol. The authors suggested that using 40% ethanol can lead to a significant increase in the ferulic acid and rutin contents, while 40% ethanol and sugar could extract the highest amount of rutin.

Recently, different technologies such as ultrasonic-assisted extraction (UAE) and supercritical fluid extraction by carbon dioxide were employed for enhancing the extraction yield and the antioxidant content of the prepared extracts. In an investigation, ultrasound technology has been used to extract antioxidant constituents from the husk of the walnut using ethanol as a food-grade solvent [137]. The optimal conditions were reported as a temperature of 60 °C, an extraction time of 30 min, and a 60% ethanol–water mixture as the used solvent system. Comparison of the UAE and conventional extraction showed that the TPC, ferric-reducing antioxidant power (FRAP), the radical scavenging activity of 1,1-diphenyl-2-picrylhydrazyl (DPPH), and the extraction yield of the UAE during 30 minutes were significantly higher than that the conventional extraction during 16 hours. It has been recommended that the obtained extract may be used as a substitute for synthetic antioxidants. Another research study reported the extraction of antioxidants from the husk of the black walnut and the use of supercritical carbon dioxide extraction with an ethanol modifier for the first time, and the optimal extraction conditions in supercritical carbon dioxide were found to be at a temperature of 68 ° C and 20% ethanol [83].

The extract of green walnut husk was also prepared with maceration and Soxhlet extraction methods, and the phenolic compounds content and antioxidant activity were determined by Folin–Ciocalteu (F-C) and DPPH radical scavenging methods [138]. The amounts of TPC and TFC for the maceration method reported by the authors were 17.81 mg gallic acid equivalents (GAEs)/g sample and 1.59 mg catechin equivalents (CEs)/g sample, respectively; for the Soxhlet method, the amounts of TPC and TFC were 98.07 mg GAEs/g sample and 38.7 mg CEs/g sample, respectively. It has been concluded that the Soxhlet extraction method leads to higher levels of polyphenols than the maceration method. Finally, it should be mentioned that the selection of a proper solvent is a key point along with the employment of a congenial extraction technique. The ‘like dissolves like’ principle is relevant for the selection of solvents. Consequently, polar solvents will extract out polar compounds, and non-polar substances can be extracted out by non-polar solvents. The most conventional technique of extraction is solvent extraction. Owing to their expanded polarity range, a hydroalcoholic solvent mixture (a mixture of alcohol and water in different ratios) is usually considered to provide great extraction yields. The improvement of sample preparation procedures has notable successes beyond standard techniques in terms of decreasing the organic solvent loss and reducing the degradation of the sample. Additionally, it results in the removal of unwanted and insoluble elements from the extract. Microwave-assisted extraction (MAE), UAE, supercritical fluid extraction (SFE), solid phase microextraction (SPME), Soxhwave, etc. [139] are considered as new techniques in recent years.

## 8. Antioxidant Content and Radical Scavenging Activity of Walnut Husk Extract

Today, some spectroscopic and colorimetric methods are available for the measurement and determination of the antioxidant content of different plant-based materials [140]. According to the ample evidence, there is a relation between the antioxidant content and its bioactivity [28,89,93,94,95,141]. In this respect, the TPC, TFC, total flavanol content (TFAC), total flavonol content (TFOC), total hydroxybenzoic acid content (THBAC), total hydroxycinnamic acid content (THCAC), TCTC, total ellagitannin content (TETC), and total gallotannin content (TGTC) of the walnut green husk were determined in different investigations, and the reported values are summarized in Table 1. There are some differences in the registered values, which can be related to the used sample materials, walnut genotypes, geographical conditions, the employed extraction solvent, and the method of extraction. The TPC for the prepared extracts derived from the walnut husk was in the range of 74.08 to 166.44 mg/g [30,31,33,134,136,142]. Meanwhile, the content of total phenolic in the range of 6.95 to 36.10 mg/g has been found for the walnut husk [26,28,83,137]. It is clear that the content of total phenolics in the prepared extracts is much higher than that of the walnut husk without any treatment. Similarly, it has been determined that the content of total flavonoids in the walnut husk extract was 22.91 to 65.2 mg/g [31,33,136], and it is higher than that of the walnut husk (0.71 to 17.81 mg/g) [28,135,138]. The data on the TFAC, TFOC, THBAC, THCAC, TCTC, TETC, and TGTC of the walnut green husk are only limited to the studies of Rywaniak et al. [142] and Meshkini and Tahmasbi [136].

The extract of the green husk of walnut fruit has been tested for its antioxidant activity and the radical scavenging activity against some free radicals. In this way, the FRAP assay and the DPPH and 2,2′-azinobis(3-ethylbenzothiazoline)-6-sulfonic acid (ABTS•^+^) radicals are the most considered among them (refer to Table 1 for further details). Unfortunately, the reported values for the antioxidant and the radical scavenging activity of the walnut husk were expressed in different units, and thus, the antioxidant activity and the antiradical properties regarding walnut husk extract are not explicit. The DPPH radical scavenging activity of the walnut husk has been comprehensively evaluated in different studies, and similar results have been reported in some of them. For example, the values for the effective concentration of 50% (EC_50_) as mg/mL in the range of 0.15 to 0.35 were reported in the studies of Oliveira et al. [134], Fernández-Agulló et al. [30] and Noshirvani et al. [138]. Other research groups examined the DPPH radical inhibition of the walnut husk and reported the values in the form of the inhibitory concentration of 50% (IC_50_) (µg/mL) [31,135,143]. The ABTS•^+^ radical scavenging capability of the walnut husk was only reported by two different research groups [135,142]. The antioxidant activity of the walnut husk has been evaluated using FRAP assay by different authors, but the obtained and reported values are different from each other [83,135,137,142,143].

## 9. Chemical Constituents of Walnut Husk

### 9.1. Hydrolysable Tannins

**Figure 2:**
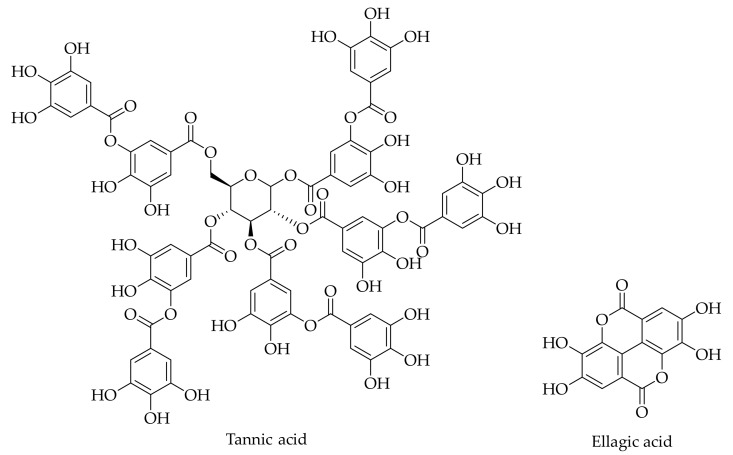
The chemical structures of different identified, isolated, and quantified hydrolysable tannins in the green husk of walnut.

Ellagic acid and tannic acid are two hydrolysable tannins that are present in the walnut husk (Table 2 and Figure 2). Ellagic acid has been detected as a hydrolysable tannin in the husk of *J. regia* L. [23,24,26,121,122]. Tannic acid is another compound that has been identified in the husk of the Persian walnut [28].

### 9.2. Naphthoquinones, Naphthoquinone Glycosides, Naphthalenes

**Figure 3:**
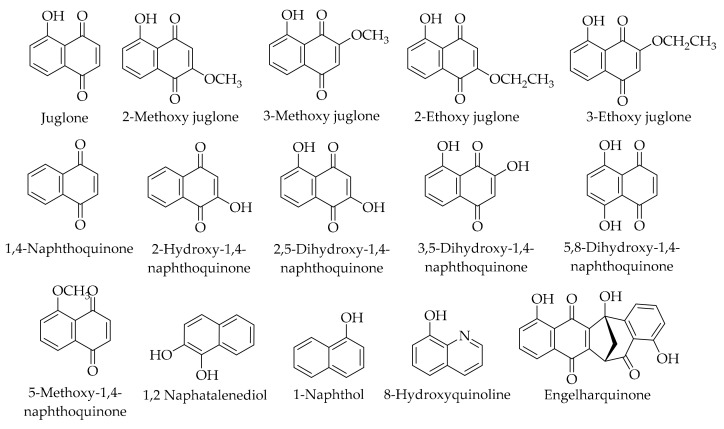
The chemical structures of different identified, isolated, and quantified naphthoquinones in the walnut green husk.

**Figure 4:**
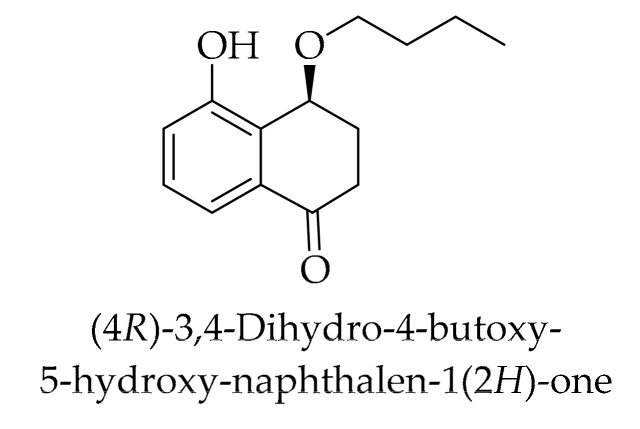
The chemical structure of new naphthalenone identified in the walnut green husk.

Naphthoquinones are a crucial group of plant chemicals that have been extensively studied in the Juglandaceae family. The retention time and the content of the corresponding naphthoquinone compounds are given in Table 2, and their chemical structures are shown in Figure 3. In a comprehensive investigation, 27 naphthoquinones and their derivatives, including four new naphthalenyl glucosides and 23 other known compounds, have been isolated from the green husk of *J. mandshurica* Maxim [144]. It has been reported that juglone was the most important phenolic compound with the highest content. This naphthoquinone was also detected in other investigations [23,24,26,121,122,136]. Recently, the 3-methoxy derivative of juglone was identified for the first time in Persian walnut husks [121]. Meanwhile, 3-methoxy and 2-methoxy juglone have been found in the green walnut husk of *J. mandshurica* Maxim [144]. Analyzing the liqueur obtained from the husk showed the presence of 1,4-naphthoquinone in Persian walnuts [26]. This naphthoquinone has been detected by other authors [24,122] in the husk of the Persian walnut. Furthermore, 1,2-naphatalenediol was also isolated from the Persian walnut husk [136], and 1-naphthol and 8-hydroxyquinoline have been quantified by HPLC in the Persian walnut husk [28]. Some derivatives of 1,4-naphthoquinone, including 5,8-dihydroxy-1,4-naphthoquinone, 2-hydroxy-1,4-naphthoquinone, 2,5-dihydroxy-1,4-naphthoquinone, 3,5-dihydroxy-1,4-naphthoquinone, 5-methoxy-1,4-naphthoquinone, and engelharquinone were identified in the husk of *J. mandshurica* Maxim [144]. Another study isolated a new naphthalenone (4*R*)-3,4-dihydro-4-butoxy-5-hydroxy-naphthalene-1 (2*H*)-one from the green husk extract of *J. mandshurica* Maxim (Figure 4) [80].

**Figure 5:**
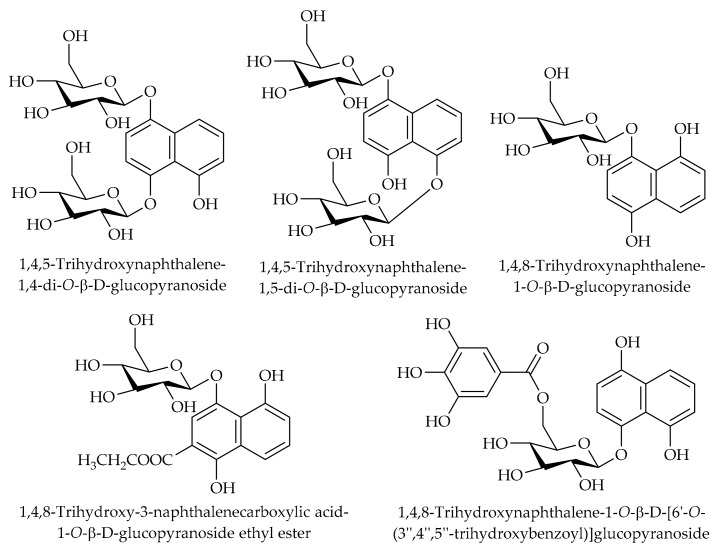
The chemical structures of different identified, isolated, and quantified naphthoquinone glycosides in the walnut green husk.

1,4,5-trihydroxynaphthalene-1,4-di-*O*-β-D-glucopyranoside, 1,4,5-trihydroxynaphthalene-1,5-di-*O*-β-D-glucopyranoside, 1,4,8-trihydroxynaphthalene-1-*O*-β-D-[6′-*O*-(3′′,4′′,5′′-trihydroxybenzoyl)]glucopyranoside, 1,4,8-trihydroxynaphthalene-1-*O*-β-D-glucopyranoside, and 1,4,8-trihydroxy-3-naphthalenecarboxylic acid-1-*O*-β-D-glucopyranoside ethyl ester have been reported as naphthoquinone glycosides in the husk of *J. mandshurica* Maxim [144]. The chemical structures of the corresponding compounds are illustrated in Figure 5.

### 9.3. α-Tetralones, α-Tetralones Glycosides, and α-Tetralone Dimers

**Figure 6:**
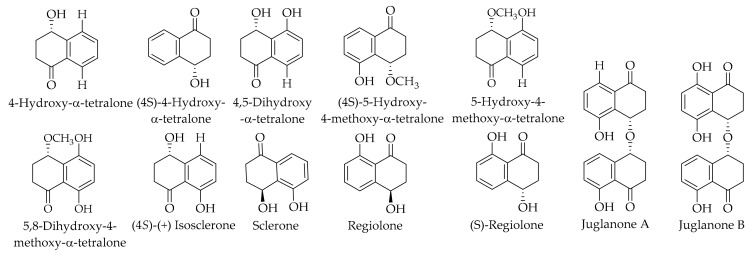
The chemical structures of different identified, isolated, and quantified α-tetralones and α-tetralone dimers in the walnut green husk.

**Figure 7:**
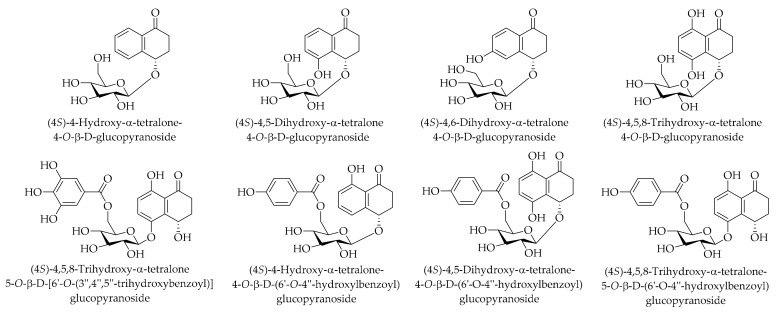
The chemical structures of different identified, isolated, and quantified α-tetralone glycosides in the walnut green husk.

Tetralones are another phytochemical group that has been widely studied in the family of Juglandaceae. Table 2 shows the retention time, and Figure 6 gives the content of the isolated and characterized compounds and their chemical structures. Du et al. [79] chemically investigated the green husk of the Persian walnut and isolated 16 different compounds. They isolated five α-tetralones, including (4*S*)-(+) isosclerone, 5,8-dihydroxy-4-methoxy-α-tetralone, (4*S*)-4-hydroxy-α-tetralone, 4,5-dihydroxy-α-tetralone, and 5-hydroxy-4-methoxy-α-tetralone, and two α-tetralone dimers—namely, juglanone A and juglanone B. The authors claimed that among the identified chemicals, compounds 5,8-dihydroxy-4-methoxy-α-tetralone, 4,5-dihydroxy-α-tetralone, and 5-hydroxy-4-methoxy-α-tetralone were isolated for the first time from *J. regia* L. green husk extract. Moreover, regiolone, along with the above-isolated compounds, is also reported as a tetralone in the husk of *J. regia* L. [121]. (*S*)-regiolone has been identified in the husks of *J. mandshurica* Maxim [144]. Sclerone was identified in the green husk extract of the Persian walnut when a rapid capillary zone electrophoresis method for the simultaneous determination of four cyclic diarylheptanoids and a tetralone derivative was developed [146]. Li et al. described the isolation of juglanones A and B as tetralone dimers with an O-bridge from the extract of the walnut husk [145]. These compounds were the first examples of O-bridged dimeric tetralones. An α-tetralone derivative (sclerone) in the extract of the Persian walnut green husk was also described [121].

(4*S*)-4-hydroxy-α-tetralone-4-*O*-β-D-glucopyranoside, (4*S*)-4,5-dihydroxy-α-tetralone 4-*O*-β-D-glucopyranoside, (4*S*)-4,6-dihydroxy-α-tetralone 4-*O*-β-D-glucopyranoside, (4*S*)-4,5,8-trihydroxy-α-tetralone 4-*O*-β-D-glucopyranoside, (4*S*)-4,5,8-trihydroxy-α-tetralone-5-*O*-β-D-(6′-*O*-4′′-hydroxylbenzoyl)glucopyranoside, (4*S*)-4-hydroxy-α-tetralone-4-*O*-β-D-(6′-*O*-4′′-hydroxylbenzoyl)glucopyranoside, (4*S*)-4,5-dihydroxy-α-tetralone-4-*O*-β-D-(6′-*O*-4′′-hydroxylbenzoyl)glucopyranoside, and (4*S*)-4,5,8-trihydroxy-α-tetralone 5-*O*-β-D-[6′-*O*-(3′′,4′′,5′′-trihydroxybenzoyl)] glucopyranoside have been reported as α-tetralone glycosides in the husk of *J. mandshurica* Maxim [144]. For more details regarding the chemical structures of the identified compounds, see Figure 7.

### 9.4. Hydroxybenzoic Acids

**Figure 8:**
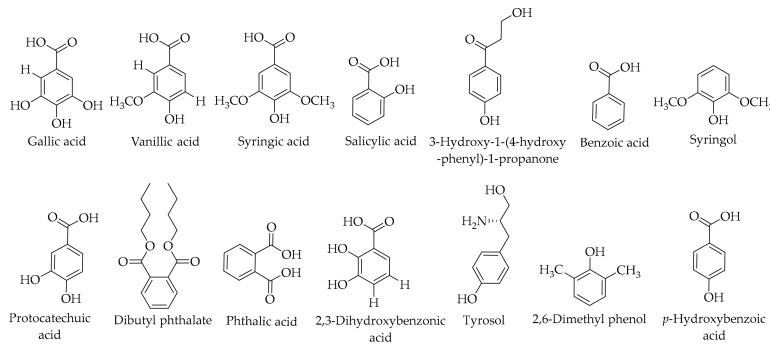
The chemical structures of different identified, isolated, and quantified hydroxybenzoic acids in the walnut green husk.

Stampar et al. [26] investigated the phenolic composition of the Slovenian walnut husk, Elit cultivar, which is traditionally used for liqueur preparation from the green husk of walnut fruit. Using the HPLC method equipped with a photodiode array (PDA) detector, they identified 13 different phenolics. Gallic acid, protocatechuic acid, syringic acid, and vanillic acid were reported as the significant hydroxybenzoic acids in the prepared walnut liqueur. The effect of solvent concentration and the addition of sugar on the properties of green walnut extracts were investigated by the preparation of three variants of extracts, reporting similar phenolic compounds in the walnut green husk extracts. In this study, 17 individual phenolics were detected and quantified by using HPLC in walnut green husk fruit extracts. Also, researchers reported the presence of salicylic acid [23]. The same compounds were also found by Rahmani et al. [33]. Du et al. detected 3,4-dihydroxybenzoic acid and 2,3-dihydroxybenzoic acid [79]. Chen et al. also isolated the first one [80] from *J. mandshurica* Maxim. husk extract. It has been claimed that the second compound was reported for the first time in the Juglandaceae family. Dibutyl phthalate is considered as another aromatic compound that is present in *J. mandshurica* Maxim husk [80]. Tyrosol and 3-hydroxy-1-(4-hydroxy-phenyl)-1-propanone were reported for the first time in the family of Juglandaceae [121]. Benzoic acid, 2,6 dimethyl phenol, syringol, and phthalic acid have been identified in the husk extract of *J. regia* L. [136]. (For further details, see Table 3 and Figure 8).

### 9.5. Hydroxycinnamic Acids

**Figure 9:**
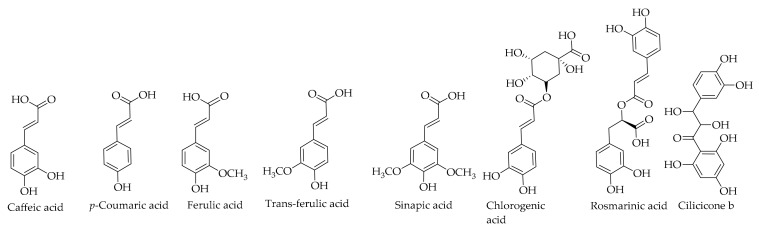
The chemical structures of different identified, isolated, and quantified hydroxycinnamic acids in the walnut green husk.

Table 4 shows the contents of the identified hydroxycinnamic acids in the green walnut husk and the corresponding chemical structures are illustrated in Figure 9. Du et al. [79] reported the isolation of caffeic acid and ferulic acid for the first time from the pericarp of Persian walnut along with 16 other compounds. These compounds have been isolated by Stampar et al. [26] and Cosmulescu et al. [23]. Additionally, chlorogenic acid, *p*-coumaric acid, and sinapic acid have been detected in the Persian walnut green husk extract [23,24,26,33,121]. Rosmarinic acid was also isolated for the first time from *J. regia* L. by Tsasi et al. [121]. Also, the same research group reported cilicicone b and trans-ferulic acid [121].

### 9.6. Flavonoids

**Figure 10:**
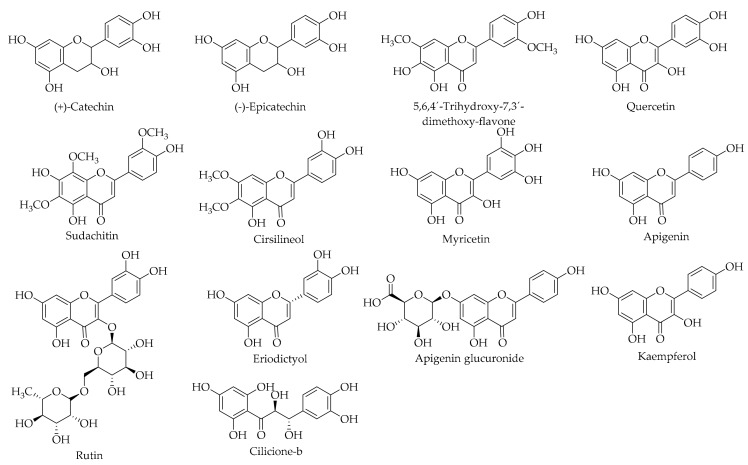
The chemical structures of different identified, isolated, and quantified flavonoids in the green husk of walnut.

The performed studies on Persian walnut green husk indicated that (+)-catechin [23,24,26], (−)-epicatechin [23,26], myricetin [23,26], and quercetin [23] are the main compounds present in walnut husk belonging to the flavonoid group of polyphenol compounds (see Table 5 and Figure 10 for more details). Regarding the content of flavonoids in the husk of *J. regia* L., the presence of different flavonoid compounds—namely sudachitin, cirsilineol, and 5,6,4´-trihydroxy-7,3´-dimethoxy-flavone—were all isolated for the first time in the Juglandaceae family, while apigenin, its glucuronide derivative, and eriodictyol were previously reported in the family, but eriodictyol has been reported for the first time in *J. regia* L. [121,147]. Meshkini and Tahmasbi [136] reported the presence of kaempferol in the husk extract of *J. regia* L. Rutin is another flavonoid compound that were reported in the husk of Persian walnut [23,33].

### 9.7. Diarylheptanoids

**Figure 11:**
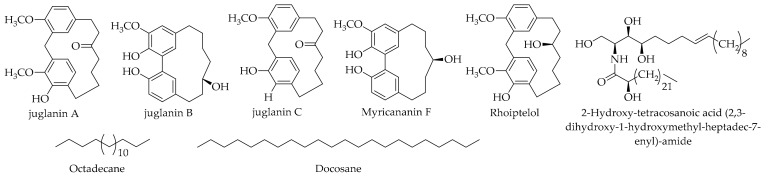
The chemical structures of different identified, isolated, and quantified diarylheptanoids, ceramides, and alkanes in the green husk of walnut.

Du et al. considered the secondary metabolites in the husk of *J. regia* L. [79], and their chemical investigation led to the isolation of 16 various compounds. The authors identified juglanin B as a diarylheptanoid. They also claimed that among the identified chemicals, juglanin B was isolated from *J. regia* L. for the first time. Juglanin B was also reported by Tsasi et al. [121] in *J. regia* L. husk extract. Li et al. isolated four diarylheptanoids (rhoiptelol, juglanin A, juglanin B, and juglanin C) [146] during the development of a rapid capillary zone electrophoresis method for the real-time determination of cyclic diarylheptanoids in *J. regia* L. green husk extract. The isolation of a diarylheptanoid myricananin F from the green husk walnut of *J. mandshurica* Maxim was also reported in the literature [80] (refer to Table 6 and Figure 11 for more details).

### 9.8. Ceramides

For the first time, 2-hydroxy-tetracosanoic acid (2,3-dihydroxy-1-hydroxymethyl-heptadic-7-enyl)-amide as a ceramide compound was isolated from the *Juglans* genus in a phytochemical study of *J. mandshurica* Maxim green husk [80] (refer to Table 6 and Figure 11 for more details).

### 9.9. Alkanes

The isolation of one alkane (octadecane) from *J. mandshurica* Maxim green husk has also been reported [80]. The presence of docosane as the other alkane compound has been described in the Persian walnut husk extract [136] (Table 6 and Figure 11).

### 9.10. Steroids

**Figure 12:**
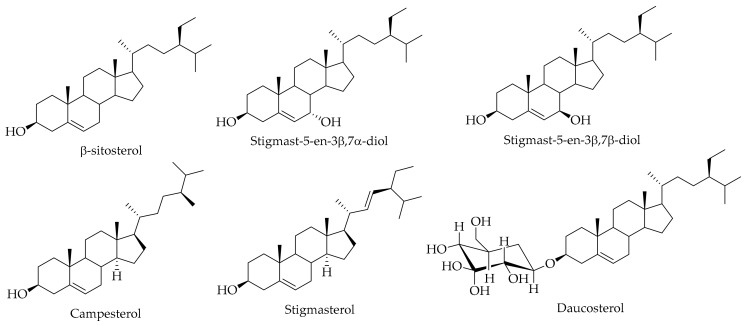
The chemical structures of different identified and isolated steroids in the green husk of walnut.

Four different steroids, namely β-sitosterol, stigmast-5-en-3β,7α-diol, stigmast-5-en-3β,7β-diol, and daucosterol (refer to Table 6 and Figure 12) were isolated [80]. The authors reported the compounds stigmast-5-en-3β,7α-diol, stigmast-5-en-3β,7β-diol, and daucosterol for the first time from the genus of *Juglans*. Campesterol and stigmasterol are two other steroids that have been identified in *J. regia* L. green husk [136].

### 9.11. Triterpenoids

**Figure 13:**
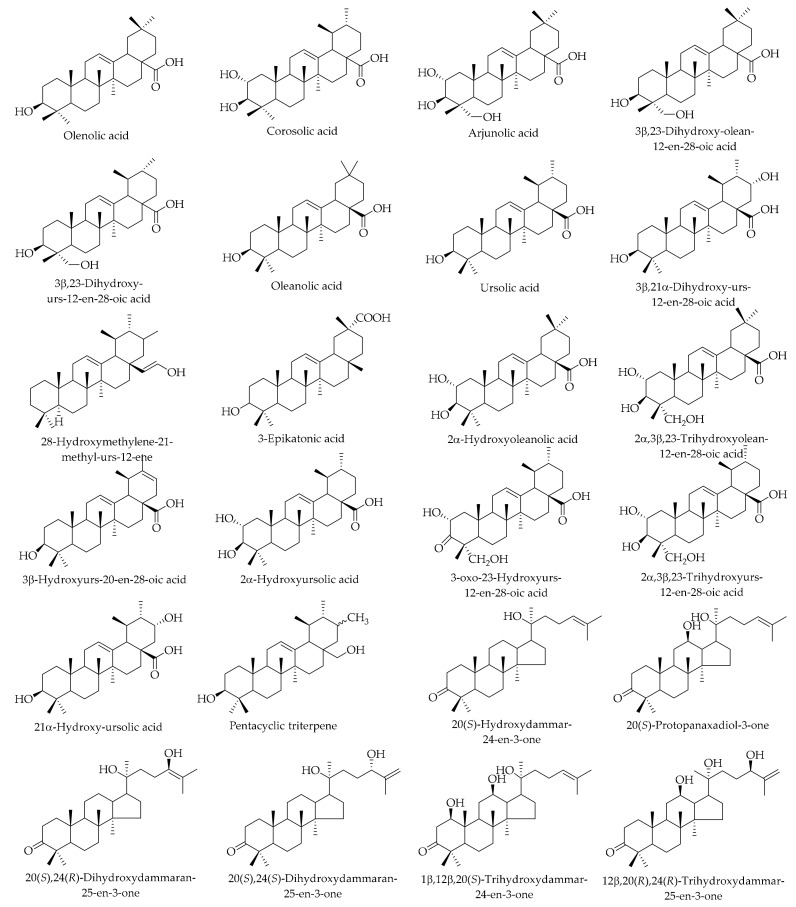
The chemical structures of different identified, isolated, and quantified triterpenoids in the green husk of walnut.

Triterpenoids are one of the largest families among the identified natural products, which have been extensively studied for their various structures and biological activities, including antitumoral activities. Zhou et al. performed extensive phytochemical research related to the *J. mandshurica* Maxim green husk [148]. This investigation reported the isolation of a new dammarane triterpene, 12β, 20(*R*), 24(*R*)-trihydroxydammar-25-en-3-one, with 16 known compounds, which were mostly from the chloroform and ethyl acetate extracts. Based on their structural properties, the authors divided the identified compounds into dammarane-type, oleanane-type, and ursane-type. They indicated that the dammarane-type triterpenoids were isolated for the first time from the Juglans genus. Additionally, the phytochemistry investigation of green husk from *J. mandshurica* Maxim resulted in the isolation of 17 different compounds and five triterpenoids (olenolic acid, corosolic acid, arjunolic acid, 3β,23-dihydroxy-olean-12-en-28-oic acid, and 3β,23-dihydroxy-urs-12-en-28-oic acid) (see Table 6 and Figure 13 for more details). The compounds 3β,23-dihydroxy-olean-12-en-28-oic acid and 3β,23-dihydroxy-urs-12-en-28-oic acid for the first time were isolated from the genus *Juglans* [80]. Phytochemical investigation of the obtained extracts with a different polarity from the green husk of *J. regia* L. in Greece yielded 32 different compounds. Four pentacyclic triterpenes—oleanolic acid, ursolic acid, 3β, 21α-dihydroxy-urs-12-en-28-oic acid, and 28-hydroxymethylene-21-methyl-urs-12-ene—were described in Persian walnuts. 3β, 21α-dihydroxy-urs-12-en-28-oic acid, and 28-hydroxymethylene-21-methyl-urs-12-ene were reported for the first time in the Juglandaceae family [121]. In another research by the same authors [147], three α-amyrine type triterpenes (ursolic acid, 21α-hydroxy-ursolic acid, pentacyclic triterpene) in addition to oleanolic acid were separated from the dichloromethane extract of the husk of *J. regia* L. It has been indicated that pentacyclic triterpene is a new natural product.

### 9.12. Sesquiterpenes

**Figure 14:**
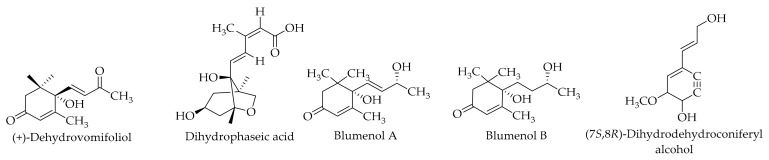
The chemical structures of different identified, isolated, and quantified sesquiterpenes and neolignans in the green husk of walnut.

Du et al. [79] isolated and reported (+)- dehydrovomifoliol as a sesquiterpene in the pericarps of Persian walnuts. In this study, (+)- dehydrovomifoliol was reported for the first time from the Juglandaceae family. For the first time, a sesquiterpene—namely, (+)-dehydrovomifoliol—was isolated from the green walnut husk of *J. mandshurica* Maxim [80]. Additionally, the separation of three sesquiterpenes—dihydrophaseic acid, blumenol A, and blumenol B—were described, and the compound Blumenol B was reported for the first time in the Juglandaceae family [121] (refer to Table 6 and Figure 14 for more details).

### 9.13. Neolignans 

The neolignan (7*S*, 8*R*)-dihydrodehydroconiferyl alcohol (see Table 6 and Figure 4) has been identified for the first time in the Juglandaceae family [121].

### 9.14. Vitamins

Two vitamins, ascorbic acid [28] and α-tocopherol [136], have been reported in the Persian walnut green husk. The content and the retention times of the corresponding compounds are summarized in Table 6.

### 9.15. Other Compounds

The presence of other compounds such as octadecanoic acid, rhodopsin, megastigma, and cyclodecasiloxane has also been reported in the green husk extract of Persian walnut [136]. Table 6 shows the content and the retention times of those compounds.

## 10. Walnut Husk Uses

**Figure 15:**
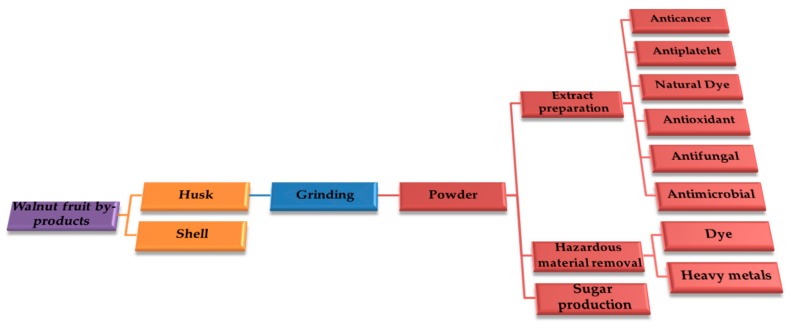
Schematic illustration of walnut fruit by-products, as well as the processing and main applications of walnut green husk.

Today, different applications of walnut husk in industry, food, and medical fields have been reported in the literature (Figure 15). The removal of hazardous materials, including the elimination of synthetic dyes and heavy metal ions from industrial effluents, are the main uses of walnut husk in the form of fine powder. For medical and food uses, the preparation of walnut husk extracts and the characterization of the bioactive compounds are necessary. For the prepared extracts and the isolated chemical constituents, excellent antioxidant, antimicrobial, antifungal, anticancer, and antiplatelet activities have been described. In the following, different uses of walnut green husk are discussed in greater detail.

### 10.1. Industrial Uses

#### 10.1.1. Removal of Hazardous Materials

##### Dye Removal

Recently, many plant-based biomaterials—in particular, waste agricultural products—have been widely employed for the removal of hazardous materials, contaminating the environment as a result of industrial activities [149]. Walnut husk has been used as an inexpensive agricultural solid waste for the removal of such dangerous compounds. The use of walnut husk as a sorbent for the removal of synthetic dyes or other hazardous compounds is reported in some studies. For example, some have tried to evaluate the kinetic aspect of Basic Red 46 (BR 46) removal by walnut husk [150]. For this purpose, artificial neural network (ANN), gene expression programming (GEP), logistic and pseudo-second-order kinetic models have been designed to predict the efficiency of Basic Red 46 (BR 46) removal on the husk of the walnut. It has been recommended that in the removal of the studied dye, functional groups such as the hydroxyl, carbonyl, and carboxyl groups in the walnut husk play an important role. Thermodynamic parameter results indicated that this process is feasible, endothermic, and spontaneous, and the maximum sorption is 66.45 mg/g. According to the ANN results, the most efficient parameter was the contact time, followed by the initial dye concentration. The results of this investigation revealed that the walnut husk was very capable of removing BR 46 from aqueous solution under different environmental conditions, and in the design and scale up, ANN and GEP models can be used to remove BR 46 from the walnut husk.

In another study reported by the same group [151], in order to predict the efficiency of the Lanaset Red G removal on the walnut husk, they used an ANN model and found that ANN was the most suitable model to describe the sorption process, based on error analysis and the determination of coefficients. ANN results showed that pH was the most significant parameter (43%), followed by the initial dye concentration (40%) for the sorption of Lanaset Red G on the husk of the walnut.

Walnut green husk as adsorbent was also examined for the removal of phenol from water [152]. The authors achieved the maximum sorption at pH 4.0, and the results indicated that the Langmuir isotherm was the appropriate model for describing the obtained data for the adsorption of phenol onto the green husk of walnut fruit. The Langmuir isotherm achieved the maximum adsorption capacity of the walnut husk for the removal of phenol by 17.8 mg/g. Besides, sorption rates were found to be consistent with pseudo-secondary kinetics with good correlation.

##### Heavy Metal Removal 

The worldwide water contamination by heavy metals is known as a crucial issue, because it causes the environmental and ecological problem threatening the life of living organisms, particularly human [153,154]. Many agricultural by-products were examined for the removal of heavy metals from wastewater as an adsorbent because of their low cost and easy availability. The walnut husk has also been used as an effective adsorbent for the removal of heavy metals. However, the reports are limited to several studies. Among different heavy metals known as hazardous metal ions, only chromium (Cr) and cadmium (Cd) have been investigated to remove using walnut husk.

Among at least 20 heavy metals known as hazardous materials, Cr is a joint surface and groundwater pollutant. Various industrial activities, such as the preservation of timber, leather tanning, textile dyeing, and electroplating are the significant causes of water contamination by Cr ions [155]. In an investigation by Wang et al. [156], they considered the removal of Cr (VI) from aqueous solution by the walnut husk. They indicated that the Cr separation was pH-dependent and the maximum of removal (97.3%) was achieved at pH 1. The authors reported that the experimental data kinetics have been well adapted to the first order, modified Freundlich, intraparticle diffusion, and Elovich models. In this investigation, the authors showed that the walnut husk is very good for the practical application of Cr removal.

In another research, the batch experiments were also conducted for the removal of aqueous Cr (VI). The obtained results revealed that the removal mechanism could be the chemical reduction of Cr (VI) to Cr (III) followed by adsorption or surface precipitation. The removal efficiency of 95% was obtained when the operational conditions for Cr (VI) removal from 50 mL of Cr (VI) = 10 mg/L solution was optimized at pH = 3.6, time = 5 min, walnut husk concentration = 6 g/L, and the ionic strength = 0.1 M. The results of this study showed that the walnut husk was affordable, effective, and suitable for removing aqueous Cr (VI), and therefore was a low-cost water treatment material [157].

Cd (II) is another heavy metal that researchers have tried to remove from the walnut green husk as a natural plant-based biosorbent. In this investigation, batch experiments were used to study the removal of Cd (II) ions by the walnut husk [158]. It was reported that the process of adsorption was pH-dependent, and the maximum adsorption at pH 7 was achieved. The adsorption of Cd (II) on the studied adsorbent could be well adapted to Langmuir and Freundlich isotherms. The optimum adsorbent concentration was 0.8 g/L of the sample solution with the initial Cd (II) concentration of 1.5 mg/L. A removal efficiency of 96.11% for Cd (II) was obtained under optimum conditions.

#### 10.1.2. Natural Hair Dye

Walnut green husk can be used as a cost-effective, valuable, environmentally friendly, and safe source of cosmetic dyeing and antimicrobial agents. To this end, the effective use of walnut green husk extract as a natural hair color has been evaluated. The coloring properties, fastness, and antimicrobial behavior of the colored hair and also a skin irritation test for natural hair color on the skin of the rat were tested. When the obtained extract was added to ferrous sulfate as a mordant agent, ascorbic acid as a developer, a cosmetic ingredient, and also Aloe vera extract as a secondary mordant, a dark brown color was observed on the examined hair samples. The colored hair showed adequate color strength with excellent morphology for a hair surface covered with color molecules. The colored hair also had excellent resistance to washing and daylight fastness, without any irritating properties as shown in a rat model, although high concentrations of iron-based mordant may be problematic for long-term use. Researchers have also been suggested the use of natural mordants such as lactic and oxalic acids to avoid potential risks [29].

### 10.2. Food Uses

#### 10.2.1. Natural Antioxidants

Antioxidants show high capacity in the protection of oils and foods against oxidation. Recently, it has been an increasing tendency among food scientists to replace synthetic antioxidants with the natural ones due to safety concerns [159]. The most used synthetic antioxidants are harmful to health, but natural antioxidants are generally supposed to be safe [160,161]. Due to the high antioxidant effects of the walnut husk, it could be the right choice for the extraction of natural antioxidants. Accordingly, the antioxidant effects of the extract and the powdered green walnut husk on the oxidation of sunflower oil have been investigated [138]. Antioxidant effects of different concentrations (100 mg/kg, 250 mg/kg, 500 mg/kg, and 1000 mg/kg) of green husk extract and powder (500 mg/kg and 1000 mg/kg) were compared with the control and artificial antioxidant tert-butylhydroquinone (TBHQ) in the maximum limit amount (200 mg/kg) through the evaluation of acidity, peroxide value, and thiobarbituric acid (TBA) on days 0, 5, 10, and 15 of storage at the temperature of 70 °C. The results showed that the peroxide value and TBA oxidation rate increased over time, but the samples containing the extract and the powder of walnut husk showed less oxidation compared to the control in most concentrations. The use of high concentrations of green walnut husk extract increased oil oxidation, and the best result was obtained for 100 mg/kg, which could well slow the oxidation process and compete with TBHQ at 200 mg/kg. This study suggested that green walnut husk with high antioxidants and a low price could be considered as a source to replace with synthetic antioxidants.

In the meat processing industry, the green husk of walnut can be used as a functional additive which is a low-cost source of valuable phytochemicals. The effect of adding green husk onto the selected properties of cooked sausages has been assessed [162], and it has been shown that the walnut husk reduced weight loss in the cooked sausage storage. Also, when the walnut husk was used in meat products, there was less color deterioration during storage. Also, the hardness of cooked sausages increased along with the addition of walnut husk, while springiness and chewiness decreased. It was found that incorporating walnut husk into sausage improved the sensory acceptance of smell and texture, and the growth of microorganisms was inhibited during the storage of cooked sausages.

#### 10.2.2. Walnut Husk Liqueur

For many years, just before the hardening of the endocarp, the green unripe walnut fruit would be picked, and after slicing, it would be left to steep in food-grade ethanol alcohol to make a delicious beverage called walnut liqueur. In Italy, “Nocino” is the name of a similar alcoholic drink that is prepared from green young walnut fruit [22]. In traditional folk medicine, the fresh green walnuts are extensively used for the preparation of walnut liqueur. This alcoholic beverage is rich in phenolic compounds and vitamins made from walnut fruit with green husks [26]. Jam and liqueur from the immature green walnut fruit are among the most commonly used food preparation recipes. Traditional walnut liqueur as a drink is commonly used in folk medicine, which is made of fresh green walnuts [23,27]. Walnut liqueur is a dark brown, bitter, and tasty drink that is often used as an aperitif or sometimes used to treat aches in the stomach. The content of phenolic compounds affects food and beverage astringency and bitterness [24]. Recently, the walnut liqueur has been receiving increasing interest in some research studies. For example, a study showed that the traditional walnut liqueur could be considered as a cocktail of phenolics [26]. Researchers have also observed the strong influence of cultivar selection and time of picking on the phenolic content of walnut liqueur [24]. Due to the traditional way in which the liqueur is made, the concentrations of individual phenolics in liqueur may be relatively low compared to the green husk [26]. It has been found that with the increasing ethanol concentration in walnut liqueur, the content of total phenolics and certain individual phenolic compounds (protocatechuic, sinapic and p-coumaric acids, and 1,4-naphthoquinone) increased [25]. The identification of 14 different phenolic compounds in walnut liqueur (chlorogenic acid, caffeic acid, ferulic acid, sinapic acid, gallic acid, ellagic acid, protocatechuic acid, syringic acid, vanillic acid, catechin, epicatechin, myricetin, 1,4-naphthoquinone, and juglone) has been reported [26]. In an investigation, the content of 10 phenolic compounds including gallic, protocatechuic, ellagic, chlorogenic, syringic, *p*-coumaric, sinapic acids, catechin, 1,4-naphthoquinone, and juglone has been quantified [24]. It was considered that the antioxidant potential of walnut liqueur and the antioxidant activity was directly correlated with the TPC, and this characteristic did not change even for many years during storage. The authors found that phenolic content has been influenced by fruit ripeness. However, the effects of temperature and the length of soaking on the liqueur phenolic composition of the fruits in ethanol were not significant [22].

### 10.3. Medical Uses

#### 10.3.1. Antimicrobial Activities

Naphthoquinones have important biological activities and are present in considerable amounts in the residue of walnut husk. Maleita et al. [122] aimed to assess the effects of pure naphthoquinones including juglone, 1,4-naphthoquinone, and plumbagin on the root-knot nematode *Meloidogyne hispanica* second-stage juvenile (*M. hispanica* J2) mortality in order to explore their potential as synthetic nematicides alternatives. Extracts were prepared and characterized from *Juglans* spp., and the authors evaluated the effects of extracts on the attraction and life cycle of *M. hispanica*. They reported that the most active compound was 1,4-naphthoquinone, which caused 42% J2 mortality at 50 ppm. They also observed that the dried husk extract was repellent and reduced the penetration of the nematode root, but it did not affect reproduction. The authors suggested that walnut husk could be used as renewable sources of products based on naphthoquinones and potentially used as bionematicides against *Meloidogyne* spp.

In the past recent years, nanoparticles (NPs) have received increased significant attention because of their many applications to different aspects of human life. A variety of methods for the synthesis of NPs have been investigated, including biogenic approaches that are both easy and environmentally friendly. The produced NPs in the presence of plant extracts exhibit unique properties that make them attractive for medical and industrial use. Thus, the development of environmentally friendly methods is necessary for their synthesis. By using walnut green husk extract, a new biological single-step method for the synthesis of silver chloride nanoparticles (AgCl NPs) at room temperature was used [32]. The macromolecules in the walnut green husk extract could act as bioreducers and stabilizers in the prepared NPs. They contained additional bioactive molecules on their surface and performed apparent antibacterial activity against both gram-negative (G ^–^) and gram-positive-bacteria (G ^+^). Interestingly, the synthesized NPs showed considerable inhibitory effects against *Escherichia coli* (*E. coli*) and *Staphylococcus aureus* (*S. aureus*) clinical isolates. Altogether, the authors suggested a new promising application of walnut green husk extract with the synthesized AgCl NPs.

It has been shown that walnut green husk extract could be used in practice as an appropriate natural hair dyeing agent and exhibited maximum antimicrobial activity in comparison with semi-synthetic and commercial hair dyes [29]. The results showed that the prepared natural dye was effective against *Bacillus subtilis* (*B. subtilis*), *S. aureus, E. coli*, *Pseudomonas aeruginosa* (*P. aeruginosa*), and *Aspergillus niger* (*A. niger*).

Abedi et al. [82] investigated the effects of extracts obtained from black walnut green husk and clotrimazole on *Candida albicans* (*C. albicans*) in female rats. Their obtained results demonstrated that the growth of *C. albicans* was significantly inhibited in female rats that had been treated for one week with the prepared vaginal creams, which contained 4% of *J. nigra* extract, and had a similar effect to clotrimazole.

The antibacterial capabilities of dichloromethane, ethyl acetate, methanol, and aqueous extracts of walnut fruit endocarp and exocarp were tested against two *S. Aureus* and *Bacillus cereus* (*B. cereus*) as G ^+^ and one *E. coli* as G ^-^ bacteria, respectively. The authors observed that all the extracts had antibacterial activity against selected bacteria except for the aqueous extract. They also concluded that in the food and pharmaceutical industries, the methanol extract from walnut could be used as a natural conservant ingredient [143].

The antimicrobial activity of the walnut husk aqueous extracts has been assessed against *B. cereus*, *B. subtilis*, *Staphylococcus epidermis* (*S. epidermis*), *S. aureus* (G ^+^ bacteria), *E. coli,* and *P. aeruginosa* (G ^-^ bacteria), and the higher growth inhibition was reported for the tested G ^+^ bacteria. The potential of the green husk of walnut was demonstrated as an economical source of antimicrobial agents [30].

The antimicrobial potential of walnut husk extracts from various cultivars has been screened against G ^+^ (*B. cereus*, *B. subtilis,* and *S. aureus*) and G ^–^ [*E. coli*, *P. aeruginosa,* and *Klebsiella pneumoniae* (*K. pneumoniae*)] bacteria, and fungi [*C. albicans* and *Cryptococcus neoformans* (*C. neoformans*)]. The results of this study showed that the growth of G ^+^ bacteria was inhibited by all the used walnut green husk extracts, and among them, *S. aureus* was the most susceptible one. The minimum inhibitory concentration (MIC) of 0.1 mg/mL was reported for all the extracts [134].

#### 10.3.2. Anti-Platelet Activities

It is well known that the polyphenolic compounds originating from plants are advantageous for human health, exerting protective effects on hemostasis. They have a particular influence on blood platelets. In one study by Rywaniak et al. [142], they aimed to demonstrate the cytotoxic activities and antiplatelet effects of the husk of the Persian walnut and the flower of arnica (*Arnica montana*) extracts on blood platelets. Their results revealed that both of the studied plant extracts did not have cytotoxicity effects on blood platelets. When the extract of the *J. regia* husk was used at 7.5 mg/mL, the ADP-induced platelet aggregation in whole blood was significantly diminished, and the platelet reactivity index (PRI) at 15 mg/mL was slightly decreased.

In another in vitro study, it has been revealed that walnut husk extract at the concentration of 50 μg/mL could inhibit the platelet aggregation induced by thrombin and protein secretion by 50% without any cytotoxic effects. Furthermore, it was shown that the extract had suppressed the generation of reactive oxygen species (ROS) and the caspase activation in thrombin-stimulated platelets. In the presence of N-acetylcysteine, the increase in thrombin-induced ROS levels in platelets was inhibited, demonstrating a link between caspase activation and cellular redox status in the activated platelets. The authors presumed that the walnut green husk extract anti-platelet activity is associated with its polyphenolic compounds and antioxidant properties. They suggested that the walnut husk could be regarded as a candidate for thrombotic disorders treatment [136].

#### 10.3.3. Cytotoxic Activities

To find new antitumor agents from natural products, juglanones A and B have been isolated as two new tetralone dimers from *J. regia* L; then, seven different human cancer cell lines (A549, MCF-7, BEL-7402, HeLa, COLO205, BGC-823, and SKOV3) have been evaluated for cytotoxic effects of the isolated compounds. The IC_50_ values for the considered cell lines have been reported in the range of 0.26 to 1.67 μM [145].

Twenty-seven naphthoquinones have been isolated from *J. mandshurica* Maxim, and all of the identified compounds were evaluated for their cytotoxic activities by the 3-(4,5-dimethylthiazo l-2-yl)-2,5 diphenyl tetrazolium bromide (MTT) test on the growth of human cancer cell line HepG-2. The results of this study showed that most aglycone naphthoquinones showed better cytotoxicity in vitro than naphthalenyl glucosides with the IC_50_ values of 7.33–88.23 μM [144].

The isolation of 17 different triterpenoid compounds was reported from the husk of *J. mandshurica* Maxim, and the cytotoxic activities were also evaluated on the growth of human cancer cells line HepG-2 by the MTT experiments. The results of this investigation showed that 20(*S*)-protopanaxadiol, 2α,3β,23-trihydroxyolean-12-en-28-oic acid, and 2α,3β,23-trihydroxyurs-12-en-28-oic acid presented higher cytotoxicity in vitro with the IC_50_ values of 10.32 ± 1.13 μM, 16.13 ± 3.83 μM, and 15.97 ± 2.47 μM, respectively [148].

Three triterpenes and six flavonoid compounds have been isolated from *J. regia* L. husk, and all of the obtained compounds and the prepared dichloromethane extract were considered for the cytotoxic activities on various human cancer cell lines, including MCF-7, HCT-116, HeLa, Κ562, Raji, and THP-1. The results of this study showed that the ursolic acid and apigenin compounds had the most potent anti-cancer activity against the cancer cells evaluated. The authors found that the cytotoxic concentration of apigenin in cancer cells did not induce apoptosis in mononuclear human peripheral blood cells. Finally, they suggested that the apigenine structure can eventually serve as a leading compound for the development of novel anti-cancer drugs with limited side effects on normal cells [147].

The cytotoxicity activity of walnut green husk extract was also evaluated using gold nanoparticles (Au NPs). In this study, the Au NPs biosynthesis by using *J. regia* L. green husk extract was investigated as the stabilizing and reducing agent [163]. It has been shown that at a moderate temperature, the prepared Au NPs have a blue shift, proper distribution, and smaller size compared to those manufactured at room temperature. They recommended that the yield of reaction could be increased using the moderate temperature compared to the room temperature. This is because of the effect of temperature on the rate of reduction. According to the obtained Fourier transform infrared (FTIR) spectrum, the surface of gold ions was successfully coated with Persian walnut extract. The authors observed that at the concentration of <250 μg/mL, Au NPs did not show any cytotoxic effects against the studied 3T3 and HT-29 as normal and cancerous cell lines, respectively. It has been suggested that the dose-dependent toxicity of the produced NPs made them an appropriate candidate for their various applications in medicine.

### 10.4. Other Uses

Based on the diluted acid hydrolysis, walnut husk has been considered as a source for the production of sugars, mainly glucose. In an investigation and using the response surface methodology (RSM), the significant pretreatment variables influencing fermentable sugar production from the green husk of walnut were evaluated [164]. In another attempt, the research group investigated the use of ANN to model the conversion rate of walnut husk glucose by concentrated acid hydrolysis [165]. It has been shown that under normal conditions and processes, the fermentable sugar was achievable by aiding the concentrated acid hydrolysis. These studies confirmed that walnut green husk could be considered as an appropriate feedstock for the production of sugar during the bioethanol production process.

## 11. Conclusions

Considerable amounts of agricultural by-products rich in phenolic or other beneficial compounds are produced during the fruit harvesting process, which has gained increasing interest due to their excellent antioxidant activities. Walnuts are recognized as a significant nut in the human diet, and young green walnuts are also widely appreciated in traditional folk medicine as a wholesome alcoholic drink for making a walnut liqueur. The green husk portion is the outer layer of walnut fruit constituting a large amount of fruit. It is available in high amounts as an inexpensive waste product that is rich in phenolic compounds.

Reviewing the investigations performed on the chemical composition of walnut husk indicated that it contains different chemicals mainly belonging to the classes of triterpenoids, naphthoquinones, and α-tetralones, which have been shown as potent anticancer agents. These constituents could be introduced as novel drugs or used as lead compounds for developing new anticancer agents.

The use of walnut husk extract as a reducing or stabilizing material in the preparation of NPs could open a new horizon for the application of this plant-based material in the nanomedicine. The excellent antioxidant capacity of walnut husk could be concerning the presence of high concentrations of antioxidants in this part of the fruit. Thus, it creates the possibility of walnut husk to use as a food antioxidant or antioxidant supplementation. Therefore, it can be used in the food industry as a source of natural antioxidants and an alternative to synthetic antioxidants. Since phenolic compounds—in particular, juglone in the green husk—have allelopathic effects on many fields and horticultural crops, the direct use of green husk as mulch or compost material is restricted. For this reason, the effective use of green husk and its derivatives as a food additive still requires further research and the development of new technologies. Due to radical scavenging and the antimicrobial effects of antioxidants in the green husk, a noticeable source of compounds with health protection potential and antimicrobial activity using new extraction and purification techniques may become necessary. In order to increase the efficiency of the extraction process, alternative techniques such as microwave-assisted extraction or UAE are currently being developed. In future studies, these methods could be used to improve the walnut green husk extraction process. Advanced techniques are responsive to a high level of automation, and some parameters can be measured at a certain time. In the case of the walnut husk, sample and solvent use can be decreased by choosing the most proper techniques. In a good extraction method, extract can be obtained in a shorter period of time, and the recovery can have increased yield and more properties than those provided by standard practice. Techniques such as SFE, MAE, and UAE have been properly suited for the extraction of walnut husk heat-sensitive and unstable substances, which is not the case with the traditional approaches. The former is more promising for industrial uses due to its enhanced performance, selectivity, and specificity. Additional modifications such as a Soxhwave under reduced pressure (which will bring together benefits such as fast heating due to the microwave, the quick boiling of solvents due to reduced pressure, and the scope of solvent recovery) will in the future make the extraction of thermolabile walnut husk components extremely more effective and fast. Nevertheless, this will require conquering innovative design and manufacturing difficulties.

According to the literature review, the green husk of walnut fruit can be used to remove hazardous heavy metal ions or other toxic materials from the polluted water as the best and most successful way of utilizing this biomass, which is available in large quantities.

## Figures and Tables

**Table 1:** The obtained values for total phenolic content (TPC), total flavonoid content (TFC), total flavanol content (TFAC), total flavonol content (TFOC), total hydroxybenzoic acid content (THBAC), total hydroxycinnamic acid content (THCAC), total condensed tannin content (TCTC), total ellagitannin content (TETC), total gallotannin content (TGTC), the radical scavenging activity against 1,1-diphenyl-2-picrylhydrazyl (DPPH), 2,2′-azinobis(3-ethylbenzothiazoline)-6-sulfonic acid (ABTS), and the reported antioxidant activity of green walnut extract using ferric-reducing antioxidant power (FRAP) assay.

No.			Content	Ref
**1**	TPC		1526 ± 111 ^4^ 74.08 ± 0.02 ^13^ 108.11 ± 4.6 ^13^ 3610 ± 55 ^28^ 84.46 ^13^ 4610.00 ± 262.73 ^1^ 6.95 ± 0.21 ^24^ 166.44 ± 1.87 ^5^ 89.07 ± 0.22 ^27^ 6.27 ^27^ 95.2 ± 6.29 ^16^ 9.17 ± 0.20 ^31^ 58.66 ± 0.37 ^24^ 122.26 ± 1.34 ^13^	[26] [134] [31] [28] [30] [23] [137] [142] [138] [135] [136] [83] [143] [33]
2	TFC		22.91 ± 1.1 ^32^ 1064 ± 81 ^29^ 423.97 ± 10.37 ^2^ 17.81 ± 0.38 ^30^ 0.71 ^19^ 65.2 ± 5.53 ^17^ 49 ± 3.17 ^14^	[31] [28] [23] [138] [135] [136] [33]
3	TFAC		34.7 ± 0.8 ^6^	[142]
4	TFOC		2.3 ± 0.1 ^8^ 21.2 ± 5.53 ^19^	[142] [136]
5	THBAC		48.8 ± 1.8 ^9^	[142]
6	THCAC		4.8 ± 0.2 ^7^	[142]
7	TCTC		3.18 ^30^ 5.8 ^18^	[135] [136]
8	TETC		11.7 ± 0.3 ^10^	[142]
9	TGTC		3.5 ± 0.3 ^11^	[142]
10	Radical scavenging activity	DPPH	0.35 ^15^ 186 ± 8.1 ^25^ 0.33 ^15^ 7850.00 ± 337.55 ^3^ 56.32 ± 2.61 ^23^ 54.9 ^25^ 0.15 ± 0.0005 ^15^ 85 ± 1.6 ^20^ 0.054 ^21^ 114 ± 1.4 ^25^	[134] [31] [30] [23] [137] [135] [138] [136] [83] [143]
		ABTS	1251± 16 ^12^ 324.8 ^25^	[142] [135]
11	Antioxidant activity	FRAP	0.45 ± 0.04 ^22^ 896 ± 18 ^12^ 0.509 ^26^ 0.0710 ± 0.0022 ^21^ 705 ± 1.2 ^33^	[137] [142] [135] [83] [143]

^1^ mg GAEs/L extract; ^2^ mg QEs/L extract; ^3^ mg TEs/L extract; ^4^ mg /100 g DW; ^5^ mg GAEs/g extract; ^6^ mg CEs/g extract; ^7^ Determined by HPLC method as CAEs; ^8^ Determined by HPLC method as QEs; ^9^ Determined by HPLC method as GAEs; ^10^ Determined by HPLC method as EAEs; ^11^ Determined by HPLC method as MGEs; ^12^ µmol TEAC/g extract; ^13^ mg GAEs/g extract; ^14^ mg CEs/g extract; ^15^ EC_50_ (mg/mL); ^16^ mg GAEs/g DE; ^17^ mg CEs/g DE; ^18^ mg leucocyanidin/g extract; ^19^ mg Res/g sample; ^20^ SC_50_ (μg/mL); ^21^ mmol TEs/g sample; ^22^ mmol Fe^2+^/g DS; ^23^ Inhibition (%); ^24^ mg GAEs/g DW; ^25^ IC_50_ (μg/mL); ^26^ Abs; ^27^ mg GAEs/g sample; ^28^ mg GAEs/100 g sample; ^29^ mg CEs/100 g sample; ^30^ mg CEs/g sample; ^31^ mg GAEs/g wet sample; ^32^ mg QEs/g extract; ^33^ mmol Fe^2+^/g DE.

**Table 2:** Different identified hydrolysable tannins and naphtoquinones, naphthalenones, α-tetralones, and α-tetralone dimers, as well as their glycoside derivatives in the green walnut husk along with their retention time and content.

No.	Compound Name		RT *	Content	Ref
**1**	Hydrolysable tannins	Ellagic acid	- - 58.51 - -	98.3 ± 5.56 ^2^ 1.57 ± 0.33 ^4^ 32.19 ± 1.65 ^1^ 2.2 ^9^ 0.3 ± 0.1 ^5^	[26] [24] [23] [121] [122]
		Tannic acid	-	120.4 ± 4.19 ^2^	[28]
2	Naphthoquinones	Juglone	- - 62.73 - - - 36.95	1404 ± 96.8 ^2^ 0.51 ± 0.02 ^3^ 34.40 ± 1.33 ^1^ 245.4 ^10^ 0.4 ^7^ 49.4 ± 0.3 ^4^ 0.21 ^16^	[26] [24] [23] [144] [121] [122] [136]
		2-Methoxy juglone	-	11.8 ^10^	[144]
		3-Methoxyjuglone	- -	22 ^11^ 1.5 ^7^	[144] [121]
		2-Ethoxy juglone	-	15.2 ^11^	[144]
		3-Ethoxy juglone	-	25.3 ^11^	[144]
		1-Naphthol	-	16.5 ± 1.41 ^2^	[28]
		8-Hydroxyquinoline	-	213.9 ± 3.05 ^2^	[28]
		1,4-Naphthoquinone	- -	0.23 ± 0.03 ^3^ 36.8 ± 0.3 ^4^	[24] [122]
		5,8-Dihydroxy-1,4-naphthoquinone	-	12.1 ^10^	[144]
		2-Hydroxy-1,4-naphthoquinone	-	33.8 ^10^	[144]
		2,5-Dihydroxy-1,4-naphthoquinone	-	15.5 ^11^	[144]
		3,5-Dihydroxy-1,4-naphthoquinone	-	7.7 ^12^	[144]
		5-Methoxy-1,4-naphthoquinone	-	5.8 ^12^	[144]
		1,2-Naphatalenediol	24.84	0.01 ^16^	[136]
		Engelharquinone	-	6.8 ^12^	[144]
3	Naphthoquinone glycosides	1,4,5-Trihydroxynaphthalene-1,4-di-O-β-D-glucopyranoside	21	5.3 ^13^	[144]
		1,4,5-Trihydroxynaphthalene-1,5-di-O-β-D-glucopyranoside	23	6.1 ^13^	[144]
		1,4,8-Trihydroxynaphthalene-1-O-β-D-[6′-O-(3′′,4′′,5′′-trihydroxybenzoyl)]glucopyranoside	22	3.2 ^13^	[144]
		1,4,8-Trihydroxynaphthalene-1-O-β-D-glucopyranoside	25	4.3 ^14^	[144]
		1,4,8-Trihydroxy-3-naphthalenecarboxylic acid -1-O-β-D-glucopyranoside ethyl ester	13	4.5 ^7^	[144]
4	Naphthalenones	(4R)-3,4-Dihydro-4-butoxy-5-hydroxy-naphthalen-1(2H)-one	-	7.1 ^5^	[80]
5	α-Tetralones	Regiolone	- -	9.8 ^5^ 2.2 ^8^	[80] [121]
		(S)-Regiolone	-	75 ^11^	[144]
		5,8-Dihydroxy-4-methoxy-α-tetralone	- -	11.7 ^2^ 0.2 ^7^	[79] [121]
		4,5-Dihydroxy-α-tetralone	- -	17.2 ^6^ 8.2 ^5^	[79] [80]
		(4S)-(+) Isosclerone	- - 21.78	42.8 ^6^ 23.4 ^11^ 0.09 ^16^	[79] [144] [136]
		Sclerone	-	0.8 ^8^	[121]
		(4S)-4-Hydroxy-α-tetralone	- -	16.9 ^6^ 0.5 ^8^	[79] [121]
		5-Hydroxy-4-methoxy-α-tetralone	-	8.4 ^6^	[79]
		(4S)-5-Hydroxy-4-methoxy-α-tetralone	-	12.8 ^12^	[144]
6	α-Tetralone glycosides	(4S)-4-Hydroxy-α-tetralone-4-O-β-D-glucopyranoside	-	7.5 ^15^	[144]
		(4S)-4,5-Dihydroxy-α-tetralone 4-O-β-D-glucopyranoside	-	5.3 ^15^	[144]
		(4S)-4,6-Dihydroxy-α-tetralone 4-O-β-D-glucopyranoside	-	4.6 ^15^	[144]
		(4S)-4,5,8-Trihydroxy-α-tetralone 4-O-β-D-glucopyranoside	-	4.4 ^15^	[144]
		(4S)-4,5,8-Thihydroxy-α-tetralone-5-O-β-D-(6′-O-4′′-hydroxylbenzoyl)glucopyranoside	42	5.9 ^7^	[144]
		(4S)-4-Hydroxy-α-tetralone-4-O-β-D-(6′-O-4′′-hydroxylbenzoyl)glucopyranoside	40	3.1 ^7^	[144]
		(4S)-4,5-Dihydroxy-α-tetralone-4-O-β-D-(6′-O-4′′-hydroxylbenzoyl)glucopyranoside	38	2.8 ^7^	[144]
		(4S)-4,5,8-Trihydroxy-α-tetralone 5-O-β-D-[6′-O-(3′′,4′′,5′′-trihydroxybenzoyl)] glucopyranoside	32	4.7 ^14^	[144]
7	α-Tetralone dimers	Juglanone A	- -	4.9 ^7^ 4.9 ^6^	[145] [79]
		Juglanone B	- -	4.8 ^7^ 4.8 ^6^	[145] [79]

* Retention time (min); ^1^ mg GAEs/L extract; ^2^ mg/100 g of DW; ^3^ mg/100 mL; ^4^ mg/g; ^5^ mg/15 kg; ^6^ mg/5.2 kg DW; ^7^ mg; ^8^ mg/488 mg; ^9^ mg/321.4 mg; ^10^ mg/15.50 g; ^11^ mg/12.80 g; ^12^ mg/8.92 g; ^13^ mg/L g; ^14^ mg/0.84 g; ^15^ mg/5.70 g; ^16^ peak area (%).

**Table 3:** Different identified hydroxybenzoic acids in green walnut husk along with their retention time and content.

No.	Compound Name	RT *	Content	Ref
1	Benzoic acid	9.59	0.1 1 ^9^	[136]
2	2,6-Dimethyl phenol	11.06	0.07 ^9^	[136]
3	Gallic acid	- - 5.25 - - ~6	122 ± 10.0 ^2^ 21.7 ± 0.43 ^4^ 66.72 ± 3.07 ^1^ 23.7 ^3^ 2.1 ^8^ 8.19 ^2^	[26] [24] [23] [79] [121] [33]
4	Vanillic acid	- - 28.09 - -	21.0 ± 2.45 ^2^ - 9.53 ± 0.43 ^1^ 13.4 ^3^ 3.4 ^7^ 2.11 ^2^	[26] [28] [23] [79] [121] [33]
5	Syringol	15.38	0.11 ^9^	[136]
6	Syringic acid	- - - 37.58 - - -	17.3 ± 2.29 ^2^ 3.58 ± 0.11 ^4^ 110 ± 3.37 ^2^ 23.00 ± 1.06 ^1^ 8.1 ^3^ 7.2 ^7^ 5.7 ± 0.2 ^5^ 85.06 ^2^	[26] [24] [28] [23] [79] [121] [122] [33]
7	Salicylic acid	- 52.04	110 ± 3.37 ^2^ 186.58 ± 6.34 ^1^	[28] [23]
8	Protocatechuic acid	- - - -	23.0 ± 4.78 ^2^ 0.74 ± 0.01 ^4^ 57.9 ^3^ 5.6 ^6^	[26] [24] [79] [80]
9	*p*-Hydroxybenzioic acid	-	2.65 ^2^	[33]
10	Dibutyl phthalate	-	5.5 ^6^	[80]
11	Phthalic acid	28.93	0.14 ^9^	[136]
12	2,3-Dihydroxybenzonic acid	-	13.9 ^3^	[79]
13	Tyrosol	-	0.9 ^7^	[121]
14	3-Hydroxy-1-(4-hydroxy-phenyl)-1-propanone	-	6.6 ^7^	[121]

* Retention time (min); ^1^ mg GAEs/L extract; ^2^ mg/100 g DW; ^3^ mg/5.2 kg DW; ^4^ mg/100 mL; ^5^ mg/g; ^6^ mg/15 kg; ^7^ mg/488 mg; ^8^ mg/468.7 mg; ^9^ peak area (%).

**Table 4:** Different identified hydroxycinnamic acids (phenylpropanoids) in green walnut husk along with their retention time and content.

No.	Compound Name	RT *	Content	Ref
1	Chlorogenic acid	- - 28.90 -	15.2 ± 2.55 ^2^ 0.32 ± 0.00 ^3^ 16.20 ± 0.68 ^1^ 2.2 ^8^	[26] [24] [23] [121]
2	Caffeic acid	- - 30.95 - - -	1.87 ± 0.10 ^2^ 383 ± 15.04 ^2^ 2.20 ± 0.09 ^1^ - 2 ^6^ 2.83 ^2^	[26] [28] [23] [79] [121] [33]
3	*p*-Coumaric acid	- 45.95 - -	0.33 ± 0.01 ^3^ 18.27 ± 0.96 ^1^ 3.5 ± 0.1 ^4^ 5.63 ^2^	[24] [23] [122] [33]
4	Ferulic acid	- 49.53 - -	21.3 ± 3.69 ^2^ 24.82 ± 0.96 ^1^ 15.2 ^5^ 6.33 ^2^	[26] [23] [79] [33]
5	Trans-ferulic acid	68.63 -	2.74 ± 0.14 ^1^ 2.4 ^6^	[23] [121]
6	Sinapic acid	- - 51.63 32.63 -	99.6 ± 22.3 ^2^ 0.12 ± 0.01 ^3^ 78.63 ± 3.61 ^1^ 0.13 ^9^ 77.13 ^2^	[26] [24] [23] [136] [33]
7	Cilicicone b	24.3 -	0.2 ^10^ 0.2 ^6^	[147] [121]
8	Rosmarinic acid	-	59.5 ^7^	[121]

* Retention time (min); ^1^ mg GAEs/L extract; ^2^ mg/100 g of DW; ^3^ mg/100 mL; ^4^ mg/g; ^5^ mg/5.2 kg DW; ^6^ mg/5.1 g; ^7^ mg/4.2 g; ^8^ mg/468.7mg; ^9^ peak area (%); ^10^ mg/2.9 kg.

**Table 5:** The retention time and content of different identified flavonoids, flavanols, and flavonol glycosides in the green walnut husk.

No.	Compound Name	RT *	Content	Ref
1	(+)-Catechin	- - 18.42	47.5 ± 6.77 ^2^ 2.07 ± 0.05 ^3^ 530.80 ± 15.39 ^1^	[26] [24] [23]
2	(−)-Epicatechin	- 40.39	23.9 ± 3.02 ^2^ 350.33 ± 11.91 ^1^	[26] [23]
3	Myricetin	- 61.56	25.0 ± 10.0 ^2^ 20.76 ± 0.98 ^1^	[26] [23]
4	Quercetin	70.68	8.16 ± 0.43 ^1^	[23]
5	Sudachitin	17.7 -	16.7 ^7^ 22.1 ^4^	[147] [121]
6	Cirsilineol	29.1 -	8.2 ^7^ 8.2 ^4^	[147] [121]
7	5,6,4´-Trihydroxy-7,3´-dimethoxy-flavone	35.0 -	2.2 ^7^ 2.2 ^4^	[147] [121]
8	Eriodictyol	-	3.7 ^4^	[121]
9	Apigenin	94.2 -	17.5 ^7^ 17.5 ^4^	[147] [121]
10	Apigenin 7-O-β-D-glucuronide	20.4 -	7.9 ^7^ 7.9 ^5^	[147] [121]
11	Rutin	57.24 -	74.70 ± 3.43 ^1^ 7.17 ^2^	[23] [33]
12	Kaempferol	34.55	0.02 ^6^	[136]

* Retention time (min); ^1^ mg GAEs/L extract; ^2^ mg/100 g DW; ^3^ mg/100 mL; ^4^ mg /5.1 g; ^5^ mg/4.2 g; ^6^ peak area (%); ^7^ mg/230 mg.

**Table 6:** Different identified diarylheptanoids, ceramides, alkanes, steroids, triterpenoids, sesquiterpenes, and neolignans in green walnut husk along with the retention time and content.

No.	Compound Name		RT *	Content	Ref
**1**	Diarylheptanoids	Juglanin A	-	0.285 ± 0.001 ^7^	[146]
		Juglanin B	- - -	1.212 ± 0.018 ^7^ 49.4 ^1^ 8.2 ^3^	[146] [79] [121]
		Juglanin C	-	0.139 ± 0.001 ^7^	[146]
		Rhoiptelol	-	0.064 ± 0.001 ^7^	[146]
		Myricananin F	-	6.8 ^2^	[80]
2	Ceramides	2-Hydroxy-tetracosanoic acid (2,3-dihydroxy-1-hydroxymethyl-heptadec-7-enyl)-amide	-	9.3 ^2^	[80]
3	Alkanes	Octadecane	-	5.1 ^2^	[80]
		Docosane	7.03	0.02 ^8^	[136]
4	Steroids	β-sitosterol	-	8.5 ^2^	[80]
		Stigmast-5-en-3β,7α-diol	-	7.3 ^2^	[80]
		Stigmast-5-en-3β,7β-diol	-	7.5 ^2^	[80]
		Stigmasterol	45.21	0.09 ^8^	[136]
		Daucosterol	-	5.1 ^b^	[80]
		Campesterol	41.05	0.03 ^h^	[136]
5	Triterpenoids	Olenolic acid	-	7.3 ^2^	[80]
		Oleanolic acid	- - -	14.4 ^10^ 5.1 ^9^ 30.3 ^4^	[147] [148] [121]
		2α-Hydroxyoleanolic acid	26	3.3 ^9^	[148]
		2α,3β,23-Trihydroxyolean-12-en-28-oic acid	-	5.2 ^9^	[148]
		3-Epikatonic acid	-	18.5 ^9^	[148]
		Corosolic acid	-	8.1 ^2^	[80]
		Arjunolic acid	-	4.6 ^2^	[80]
		Ursolic acid	- - -	11.7 ^10^ 5.2 ^9^ 0.9 ^5^	[147] [148] [121]
		21α-Hydroxy-ursolic acid	-	0.9 ^10^	[147]
		2α-Hydroxyursolic acid	33	3.5 ^9^	[148]
		3β, 21α-Dihydroxy-urs-12-en-28-oic acid	-	3.3 ^5^	[121]
		3β,23-Dihydroxy-olean-12-en-28-oic acid	-	6.5 ^2^	[80]
		3β,23-Dihydroxy-urs-12-en-28-oic acid	-	7.6 ^2^	[80]
		2α,3β,23-Trihydroxyurs-12-en-28-oic acid	-	5.6 ^9^	[148]
		3-Oxo-23-Hydroxyurs-12-en-28-oic acid	-	8.2 ^9^	[148]
		3β-Hydroxyurs-20-en-28-oic acid	30	4.2 ^9^	[148]
		28-Hydroxymethylene-21-methyl-urs-12-ene	-	8.6 ^4^	[121]
		20(S)-Protopanaxadiol	-	6.4 ^9^	[148]
		20(S)-Hydroxydammar-24-en-3-one	-	23.2 ^9^	[148]
		20(S)-Protopanaxadiol-3-one	-	12.1 ^9^	[148]
		20(S),24(R)-Dihydroxydammaran-25-en-3-one	-	4.4 ^9^	[148]
		20(S),24(S)-Dihydroxydammaran-25-en-3-one	-	15.6 ^9^	[148]
		12β,20(R),24(R)-Trihydroxydammar-25-en-3-one	-	16.4 ^9^	[148]
		1β,12β,20(S)-Trihydroxydammar-24-en-3-one	-	7.3 ^9^	[148]
		1β,3α,12β,20(S)-Tetrol-24-ene-dammar	-	22.5 ^9^	[148]
6	Sesquiterpenes	(+)-Dehydrovomifoliol	-	5.3 ^1^	[79]
		Dihydrophaseic acid	-	2.8 ^6^	[121]
		Blumenol A	-	1.3 ^5^	[121]
		Blumenol B	-	0.5 ^6^	[121]
7	Neolignans	(7S, 8R)-Dihydrodehydroconiferyl alcohol	-	2.4 ^6^	[121]
8	Vitamins	Ascorbic acid	-	5.20 ^11^	[28]
		α-Tocopherol	39.35	0.21 ^8^	[136]
9	Other compounds	Octadecanoic acid	33.27	0.11 ^8^	[136]
		Cyclodecasiloxane	30.50	0.19 ^8^	[136]
		Rhodopin	22.60	0.04 ^8^	[136]
		Megastigma	18.80	0.03 ^8^	[136]

* Retention time (min), ^1^ mg/5.2 kg DW; ^2^ mg/15 kg; ^3^ mg, ^4^ mg/5.1 g; ^5^ mg/468.7 mg; ^6^ mg/321.4 mg; ^7^ mg/g DS; ^8^ peak area (%); ^9^ mg/10 kg; ^10^ mg/2.9 kg; ^11^ mg/100 g DW.

## Abbreviations

*A. niger*	*Aspergillus niger*
Abs	Absorbance
ABTS•^+^	2,2′-azinobis(3-ethylbenzothiazoline)-6-sulfonic acid
AgCl NPs	Silver chloride nanoparticles
ANN	Artificial neural network
Au NPs	Gold nanoparticles
*B. cereus*	*Bacillus cereus*
*B. subtilis*	*Bacillus subtilis*
BR 46	Basic Red 46
*C. albicans*	*Candida albicans*
*C. neoformans*	*Cryptococcus neoformans*
CAEs	Chlorogenic acid equivalents
Cd	Cadmium
CEs	Catechin equivalents
Cr	Chromium
DE	Dry extract
DPPH	1,1-Diphenyl-2-picrylhydrazyl
DS	Dry sample
DW	Dry weight
*E. coli*	*Escherichia coli*
EAEs	Ellagic acid equivalents
EC_50_	Effective concentration of 50%
F-C	Folin-Ciocalteu
FRAP	Ferric reducing antioxidant power
FTIR	Fourier-transform infrared
G ^-^	Gram-negative
G ^+^	Gram-positive
GAEs	Gallic acid equivalents
GEP	Gene expression programming
HPLC	High-performance liquid chromatography
IC_50_	Inhibitory concentration of 50%
*J. mandshurica* Maxim	*Juglans mandshurica* Maxim
*J. nigra*	*Juglans nigra*
*J. regia* L.	*Juglans regia* L.
*K. pneumonia*	*Klebsiella pneumoniae*
*M. hispanica* second-stage juvenile	*Meloidogyne hispanica* J2
MAE	Microwave-assisted extraction
MGEs	Methyl gallate equivalents
MIC	Minimum inhibitory concentration
MTT	3-(4,5-Dimethylthiazo l-2-yl)-2,5 diphenyl tetrazolium bromide
NPs	Nanoparticles
*P. aeruginosa*	*Pseudomonas aeruginosa*
PDA	Photodiode array
PRI	Platelet reactivity index
QEs	Quercetin equivalents
REs	Rutin equivalents
ROS	Reactive oxygen species
RSM	Response surface methodology
*S. aureus*	*Staphylococcus aureus*
*S. epidermis*	*Staphylococcus epidermis*
SC_50_	50% scavenging capacity
SFE	Supercritical fluid extraction
SPME	Solid phase microextraction
TBA	Thiobarbituric acid
TBHQ	Tert-butylhydroquinone
TCTC	Total condensed tannin content
TEAC	Trolox equivalent antioxidant capacity
TEAC	Trolox equivalent antioxidant capacity
TEs	Trolox equivalents
TETC	Total ellagitannin content
TFAC	Total flavanol content
TFC	Total flavonoid content
TFOC	Total flavonol content
TGTC	Total gallotannin content
THBAC	Total hydroxybenzoic acid content
THCAC	Total hydroxycinnamic acid content
TPC	Total phenolic content
UAE	Ultrasonic-assisted extraction
WS	Wet sample
